# Molecular and biochemical responses in the midgut of the silkworm, *Bombyx mori*, infected with *Nosema bombycis*

**DOI:** 10.1186/s13071-018-2755-2

**Published:** 2018-03-06

**Authors:** Zhi Li, Yu Wang, Linling Wang, Zeyang Zhou

**Affiliations:** 1 College of Life Sciences, Chongqing Normal University, Chongqing, 401331 China; 2 State Key Laboratory of Silkworm Genome Biology, Southwest University, Chongqing, 400716 China

**Keywords:** *Nosema bombycis*, Silkworm midgut, Interaction, Differential gene expression, Biochemical response

## Abstract

**Background:**

Microsporidia are a group of eukaryotic intracellular parasites that infect almost all vertebrates and invertebrates. However, there is little information available of how microsporidia obtain nutrients and energy from host cells. The purpose of this study was to investigate the energy and material requirements of *Nosema bombycis* for the invasion procedure through analyzing the global variation of the gene expression, protein abundance, fatty acids level and ATP flux induced by the microsporidia *N. bombycis* infection in the midgut of the silkworm *Bombyx mori.*

**Methods:**

A suppression subtractive hybridization (SSH) and quantitative real-time PCR (qPCR) analysis were performed to identify the genes upregulated in the midgut of *B. mori* 48 h following *N. bombycis* infection. Gene Ontology (GO) and the Kyoto Encyclopedia of Genes and Genomes (KEGG) analyses were used to annotate and summarize the differentially expressed genes, according to the categories ‘molecular function’, ‘cellular component’ and ‘biological process’. To evaluate the nutrition material and energy costs in *B.mori* infected by *N. bombycis*, biochemical analysis was performed to determine the variation of protein abundance, fatty acid levels and ATP flux with or without the microsporidia *N. bombycis* infection in the midgut of the silkworm *B. mori*.

**Results:**

A total of 744 clones were obtained, 288 clones were randomly selected for sequencing, and 110 unigenes were generated. Amongst these, 49.21%, 30.16% and 14.29% genes were involved in 19 molecular functions, 19 biological processes and nine cellular components, respectively. A total of 11 oxidative phosphorylation- and eight proton-coupled ATP synthesis-related genes were upregulated. Seven protein degradation-, three fat degradation-related genes were upregulated, and no genes related to the *de novo* synthesis of amino acids and fatty acids were significantly upregulated. The data from the biochemical analysis showed the contents of total protein and ATP of *B. mor*i midgut tissues decreased significantly, whereas the fatty acid content did not significantly change after four days of *N. bombycis* infection. Microsporidia *N. bombycis* infection upregulated the expression level of genes involved in host ATP synthesis, protein and fat degradation, which eventually causes the obvious decline of protein content and ATP synthesis in the host midgut, whereas the fatty acids content did not change significantly.

**Conclusions:**

This study suggested to some extent that *N. bombycis* invasion can activate the host protein degradation and accelerate the production of host ATP. Microsporidia of *N. bombycis* show preference for proteins rather than fatty acids from the host to ensure the material preparation required by their parasitic life-cycle. Requirements of *N. bombycis* for energy were also mainly dependent on the host ATP production. This study provides a new data that may help our understanding of the molecular mechanisms of obtaining energy and nutrients from the host by the microsporidium *N. bombycis*.

**Electronic supplementary material:**

The online version of this article (10.1186/s13071-018-2755-2) contains supplementary material, which is available to authorized users.

## Background

Microsporidia are a group of eukaryotic intracellular parasites that infect almost all vertebrates and invertebrates and cause serious human diseases and major economic losses in the livestock industry. There are no prospective drugs to counteract this pathogen. The pébrine disease caused by microsporidia *Nosema bombycis* infection is a devastating disease for sericulture. *Nosema bombycis* is one of the major quarantine pests that have been established in silk-producing countries [1]. This microsporidian has evolved a unique way to invade the host cell. First, upon appropriate environmental stimulation, *N. bombycis* extrudes the polar tube, penetrates the membrane of the host cell and enters the cytoplasm. Then, their sporoplasm is transferred into the cytoplasm of the host cells, where the spores develop and complete the life-cycle [2–4]. This injection-like method can effectively circumvent the attacks from the host's multiple defence systems. Similar to the other reported microsporidia, the genome of the obligate parasite *N. bombycis* is small and compact [5–8]. The parasites lack mitochondria and only contain a mitosome which is thought to be a relic of the mitochondria; some genes involved in energy and material metabolism have been lost [5, 9, 10]. Their production of energy and material is far from enough to facilitate the growth and reproduction. Much energy must be obtained from the host cells.

The silkworm *Bombyx mori* is the natural host of *N. bombycis*. The mechanism of the interaction between *B. mori* and its pathogens has attracted considerable attention [11–13]. Over the past few years, researchers have used Suppression Subtractive Hybridization (SSH), DNA microarray system, high throughput expressed sequence tags, and isobaric Tag for Relative and Absolute Quantization (iTRAQ) proteomics-based methods to examine changes in gene transcription in the midgut of the silkworm *B. mori*, mainly focusing on the host immune responses to exogenous pathogenic infection [14–19]. As the first directly infected organ, the midgut has an induced high level of tissue-specific gene expression. Previous research found that a total of 36 genes and 20 novel expressed sequence tags (ESTs) were altered in the midgut of *B. mori* infected by *B. mori* cytoplasmic polyhedrosis virus (BmCPV) [14]. Recently, iTRAQ-based quantitative proteomic analysis found that a putative p62/ sequestosome-1 protein in silkworm was upregulated and may play an important role in regulating the autophagy and apoptosis (especially apoptosis) after BmCPV infection [15]. In addition to the BmCPV, the latest research based on comparative subcellular proteomics analysis found that 16 proteins in larval silkworm midgut were potentially involved in repressing *B. mori* nucleopolyhedrovirus (BmNPV) infection [16]. Moreover, BmNPV invasion also causes complex protein alterations in the larval midgut, and these changes are related to the cytoskeleton, immune response, apoptosis, ubiquitination, translation, ion transport, endocytosis and endopeptidase activity [17]. In addition, pathogenic *Bacillus bombyseptieus* invasion can also induce a strong transcriptional response in the midgut, with altered transcription of genes encoding metalloproteinases, hydrolases, lipases and chitin structural proteins [18]. For pathogenic microsporidia, the only study of transcriptional responses of *B. mori* to microsporidia *N. bombycis* infection was published in 2013 [19]. Many immune-related genes involved in melanization, humoral and cellular immunity exhibited an apparently induced upregulated expression. However, some genes involved in the energy and material transport between *B. mori* and the pathogenic *N. bombycis* remain to be further addressed. So far, there is little information available of how pathogens such as *N. bombycis* obtain nutrients and energy from their host *B. mori*. The only indirect evidence showed that in vitro spore germination upregulated the expression level of some genes involved in the glycolysis, the pentose phosphate pathway, purine and pyrimidine metabolism*,* suggesting preparations of energy generation and substance synthesis for *N. bombycis* following invasion and proliferation inside the host [20].

In this study, the global variation of the gene expression, protein abundance, fatty acids level and ATP flux induced by the microsporidia *N. bombycis* infection in the midgut of the silkworm *B. mori* was investigated. Our work provides new insights into the *N. bombycis* metabolic requirements and the interaction between microsporidia and their insect host and lists candidate drug targets for the treatment of microsporidian diseases of the silkworm *B. mori*.

## Methods

### Infection protocol

The *B. mori* strain used in this study (Dazao P50) was preserved in the Southwest University Silkworm Gene Library. The *N. bombycis* CQ1 isolates were obtained from the State Key Laboratory of Silkworm Genome Biology in Southwest University and preserved by the China Veterinary Culture Collection Center (CCVC) (Accession no. CCVC102059).

*Bombyx mori* larvae were reared under conventional conditions with mulberry leaves [21]. The larvae were then randomly divided into 2 groups, with the experimental group being fed 5 μl *N. bombycis* mature spores (suspended at 1 × 10^6^ spores/ml) sprayed on the mulberry leaves; the control group was fed 5 μl sterile water. After 48 h, midgut tissues of 3 larvae from both groups were isolated, snap-frozen in liquid nitrogen, and preserved at -80 °C. The remaining larvae from each group were reared until the 4th day after infection, when the midgut was isolated for electron microscopy and the determination of the total protein and fatty acids.

### RNA isolation and cDNA preparation

Total RNA of the midguts of *N. bombycis*-infected larvae at 48 h was extracted using the RNAiso Plus kit (Takara BioTech, Dalian, China). Double-stranded cDNA was synthesized by using the SMARTer cDNA Synthesis kit (Clontech, Shanghai, China) and purified by using the CHROMA-SPIN-1000 chromatography column (Clontech). All procedures were carried out according to manufacturers’ instructions.

### *Rsa*I digestion and purification of double-stranded cDNA

The purified double-stranded cDNA was digested with *Rsa*I enzyme in *Rsa*I restriction buffer at 37 °C for 3 h, and the reaction was terminated by adding 0.2 M EDTA. The digested cDNA was purified by phenol-chloroform extraction method and dissolved in double-distilled water. The final cDNA concentration was adjusted to 300 ng/μl for subsequent experimental work.

### Construction of subtracted cDNA libraries

Subtractive hybridization was performed for the midgut cDNA products with the experimental group as the tester and the control group as the driver, followed by nested PCR. The efficiency of subtractive hybridization was analyzed based on changes in the abundance of the housekeeping gene G3PDH before and after the subtraction. The Takara DNA Fragment Purification kit (Takara BioTech, Dalian, China) was used to purify the PCR products; these were ligated with pMD19-T vector by using the Takara DNA Ligation kit (Takara BioTech, Dalian, China). The ligation product was transformed into *Escherichia coli* competent cells JMl09 (Takara BioTech, Dalian, China), spread on LB plates, and cultured overnight at 37 °C. The numbers of both blue and white colonies were counted, and 24 white colonies with 1 mm diameter from one plate were randomly selected and collected into bacteria-detection PCR tubes as templates. The PCR reactions were carried out with the universal primers targeting both ends of the T vector, M13-47 and RV-M to determine the success of the ligation and the size of the inserted fragment.

### Sequencing and bioinformatics analysis

A total of 288 positive clones with inserted fragments were selected for plasmid extraction (Takara BioTech) and sequencing (Invitrogen BioTech, Shanghai, China). The assembled sequences were analyzed by BLAST program. Other sequences from NCBI (http://www.ncbi.nlm.nih.gov/) and the silkworm genome database (http://www.silkdb.org/silkdb/) were used to identify nucleic acid homologous. Gene ontology (GO) annotation was performed for the differentially expressed genes using the Blast2GO software and the online molecular annotation system Capital Bio Corp (MAS 2.0, http://www.capitalbio.com/). Upregulated genes were further functionally analyzed using the online metabolism pathway database, the Kyoto Encyclopedia of Genes and Genomes (KEGG) (http://www.genome.jp/kegg/).

### Quantitative real-time PCR analysis

Midgut total RNA was extracted using the RNasy Protect Mini Kit (Qiagen, Shanghai, China). The RNA was diluted to 50 ng/μl for cDNA synthesis using the PrimeScriptTM RT cDNA reagent Kit (Takara BioTech). The quality of cDNA product was evaluated by gel electrophoresis. qPCR was carried out with SYBR Premix EX Taq II (Takara BioTech) in an iQ5 qPCR machine using *β-actin* as the reference gene and the primers listed in Table 1. The PCR products were determined as specific when the melting curves were single peaks. qPCR was performed for a series of dilution standards of 7 genes, and the amplification efficiency of these genes was close to 100%. qPCR reaction was repeated three times for each sample, and the mean of the three repeats was compared to the amplification of *β-actin* for quantification. qPCR products were sequenced by Shanghai Biological Engineering Technology Services Co., Ltd. to confirm whether the amplified fragments were the target sequences. For the analysis of the data and graphical representation, the 2^-△△Ct^ method was used [22]

**Table 1:** Primers used in real-time PCR

Target gene	GenBank ID	Forward primer (5'-3')	Reverse primer (5'-3')
G3PDH	DQ443421.1	CATATTAAGCTACCGTCAGA	GGAAACCAATCTTGCCGTGT
*β-actin*	BMU49854	GGATGTCCACGTCGCACTTCA	CGGAGAGGTCGCGTCCAAACA
Serine protease 1	NP_001036826	GCGGCCGAGGTACCAGGATT	CGGAGCGGGTGTTGGTCAGT
Serine protease 5	AAX39408	TGGTGCTCACCCCCATCTTGC	CGGCCGAGGTACCAAAAGCGA
Serine protease 11	AAB26023.1	TCGCGACCACCGACGTCCAA	TGGGGTGCTACATCGCTCGGA
Lipase	NP_001036966	GGTACATGGGGACGATTGCGAGC	GAACACCCTCGCCATCAGGCAT
Membrane protein	ABK23569	CGCTAGCGCCAGTGGACGTG	CGGAGAGGTCGCGTCCAAACA
Vacuolar ATP synthase subunit G	NP_001040287	GGCAGGTTTTCTTGGAGCCGA	CGGGCAGGTACCGAGCTGAT

### Protein, fatty acid and ATP analysis of the midgut tissue

For protein concentration analysis, after 4 days of *N. bombycis* infection, the midgut tissues of *B. mori* larvae from infection and control groups were isolated, weighed, homogenized in the RIPA lysis buffer (50 mM Tris, 150 mM NaCl, 1% Triton X-100, 1% sodium deoxycholate, 0.1% SDS, sodium orthovanadate, sodium fluoride, EDTA, 1 mM PMSF) and centrifuged at 12,000× *g* and 4 °C for 10 min. The supernatant was collected to determine the protein content using the classical Bradford method [23] and the Bradford Protein Concentration Kit (Beyotime, Shanghai, China), according to the manufacturer’s instructions.

For analysis of fatty acids, the pellet was dried, weighed, resuspended in chloroform - methanol - sterile H_2_O (6:12:5, v/v/v) and rotated for 14 h at room temperature. Then, chloroform - sterile H_2_O (6:6, v/v) was added, mixed rapidly and filtered. The chloroform layer was dissolved in N-hexane and KOH - methanol solvent (0.5 mol/l) with 4:1 volume ratio at 70 °C for 20 min, vibrated in the ultrasonic cleaner for 5 min, centrifuged at 3500× *g* for 10 min, and the fatty acids were collected from the N-hexane layer. Finally, quantitative fatty acid analysis was conducted by gas chromatography with mass spectrometric detection (GC-MS, Thermo Trace, Silicon Valley, USA) [24], with minor modifications. Briefly, the oven temperature was set to 40 °C for 1 min and then increased to 260 °C at a rate of 5 °C/min and holding for 10 min. The helium carrier gas was set at a flow rate of 1 ml/min. The GC inlet was held at a temperature of 230 °C. The equilibration time between injections was 0.2 min. The temperatures of the MS source and the MS quadrupole were maintained at 250 °C and 200 °C, respectively. The mass spectra were acquired in full scan mode over the mass range of 50–500 m/z. Finally, fatty acid relative content was calculated according to the peak areas by integrating the respectively extracted ion chromatograms (EIC) with Xcalibur software.

Adenosine triphosphate (ATP) content in the midgut tissues was measured using a firefly luciferase-based ATP assay kit S0026 (Beyotime), according to the manufacturer’s protocol. Midgut tissues were collected after washing in PBS and lysed in 200 μl of lysis buffer/20 mg tissue, then homogenated with a glass homogenizer. The lysates were centrifuged at 12,000× *g* for 5 min at 4 °C. The supernatants were collected and 20 μl was added to a 96-well plate with 100 μl pre-added ATP test solution followed by incubation at RT for 10 min. OD values were measured at 570 nm using a fluorescence multimode microplate reader (Tecan Infinite M200 Pro, Switzerland). The ATP content was calculated from the standard curve [25].

### Statistical analysis

The average values of biological replicates (*n* = 3) were given as the mean ± standard deviation (SE). Statistically significant differences between the infected and control non-infected for total protein, fatty acid and ATP content, and qRT-PCR determination was subjected to the Student’s t-test in the Statistical Package for Social Science (SPSS version 23.0; SPSS Inc., Chicago, IL, USA). A *P*-value of ≤ 0.05 was considered as statistically significant.

## Results

### Confirmation of infection

The midguts of *B. mori* larvae infected by *N. bombycis* for 4 days were isolated and observed under the electron microscope, which confirmed infection by *N. bombycis* compared with the control group. Also, five gene fragments identified by the subtractive library screening perfectly matched the *N. bombycis* genomic data (http://silkworm.genomics.org.cn/), demonstrating the effectiveness of the infection.

### Quality of the subtracted cDNA libraries

**Fig. 1:**
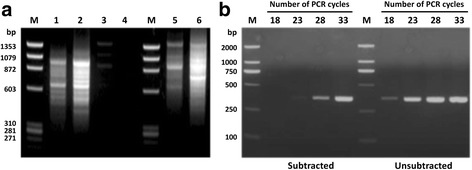
PCR evaluation of subtractive hybridization efficiency. **a** Gel electrophoresis of subtractive hybridization products after two rounds of PCR amplification. Lane M: Ф X174-*Hae* III digest Marker; Lane 1: double PCR product of subtractive hybridization group; Lane 2: double PCR product of non-subtractive hybridization group; Lane 3: positive control; Lane 4: negative control; Lane 5: double PCR product of mouse control (subtractive hybridization); Lane 6: double PCR product of mouse control (no subtractive hybridization). **b** Gel electrophoresis of *G3PDH* gene PCR products using subtractive and non-subtractive cDNA as templates with 18, 23, 28 and 33 cycles of amplification

Subtractive hybridization was performed for the midgut cDNA products with the experimental group as the tester and the control group as the driver, followed by two rounds of PCR. Analysis of the PCR products showed that the enriched bands were detectable in the subtractive group, suggesting the effective enrichment of differentially expressed genes (Fig. 1a).

As shown in Fig. 1b, the PCR product of the housekeeping gene G3PDH (glyceraldehyde-3-phosphate dehydrogenase) could be observed after 18 cycles of amplification without subtractive hybridization. Following subtractive hybridization, the amplified G3PDH was barely observed even after 28 cycles of amplification, this confirming the efficiency of the subtraction protocol.

A total of 769 colonies were generated, 744 of which were white (< 5% self-ligation rate). Twenty-four white colonies were randomly selected for PCR analysis, and 21 of them contained inserted fragments (87.5% positive colony rate). The fragment length ranged between 500–1000 bp, with most of them within the range of 600–800 bp. A total of 288 clones were randomly selected for sequencing, and 265 positive sequences were obtained after eliminating low-quality and vector sequences.

### GO and KEGG analysis of differentially expressed genes

**Fig. 2:**
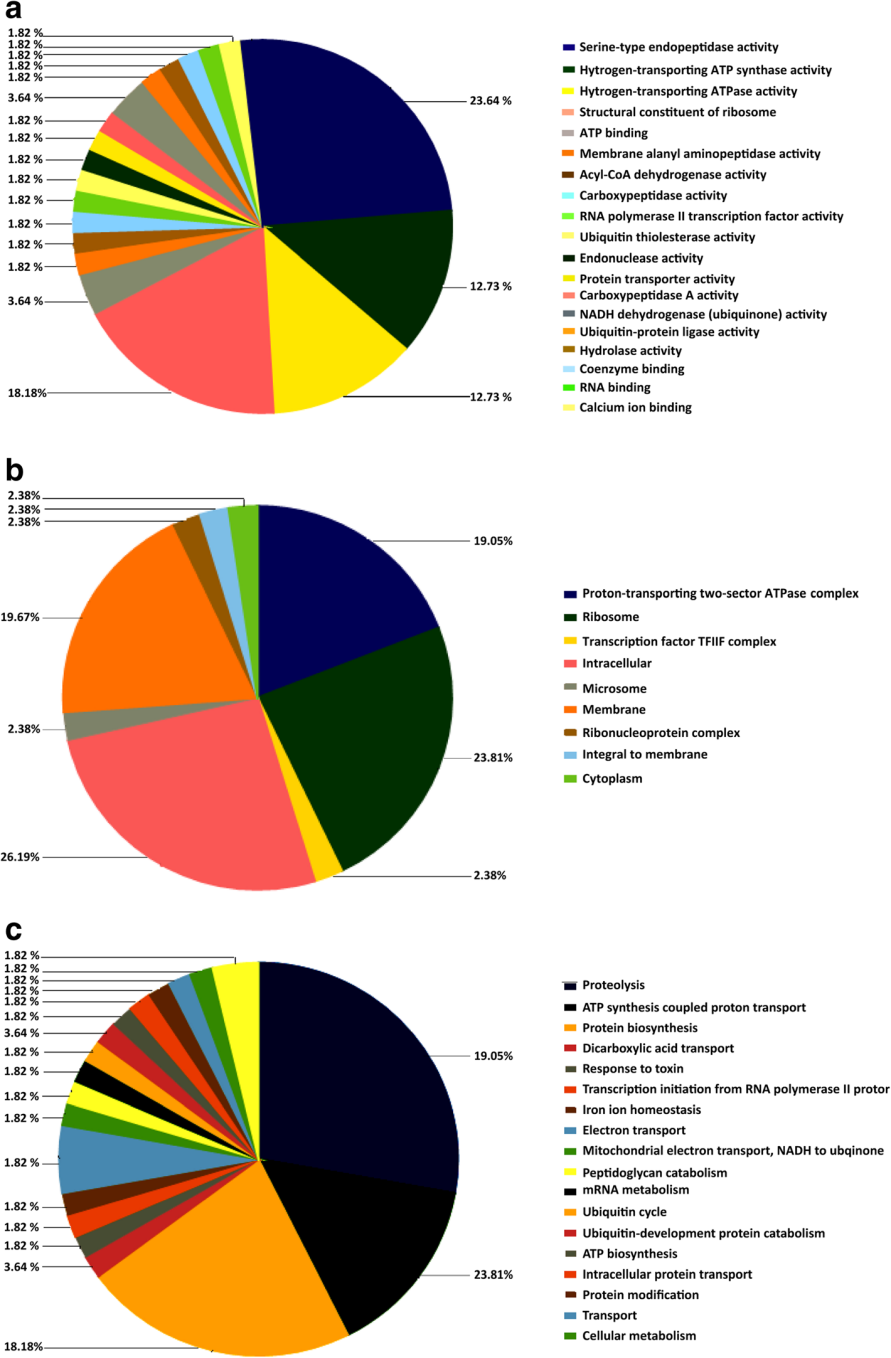
Distribution of Gene Ontology (GO) functional categories (level 2) of Suppression Subtractive Hybridization libraries for *Bombyx mori*. **a** Molecular function. **b** Cellular component. **c** Biological process. Each annotated sequence is assigned at least one GO term. Numbers refer to percentage of assigned unigenes in each category

The 265 positive sequences were aligned with BlastX, and repetitive sequences were removed. As a result, 110 sequences were annotated, 102 of which showed significant homology with genes encoding proteins with known functions in the database (Additional file 1: Table S1). GO functional analysis revealed that 49.21% of the differentially expressed genes were involved in 19 molecular functions, along with 30.16% of them playing roles in 19 biological processes and 14.29% of them being involved in 9 cellular components (Fig. 2).

In level-2 GO classification, the differentially expressed genes were mainly involved in molecular function activity (Fig. 2a) as below: serine-type peptide chain endoproteinase activity, hydrogen transport ATP activity, hydrogen transport ATP synthase activity, and cytochrome *c* oxidase activity, cytochrome *c* oxidase activity, acyl-coenzyme A binding activity, trivalent iron-binding activity, GTP binding activity, endonuclease activity, and nucleotide binding and catalytic activity. The differentially expressed genes in biological processes (Fig. 2c) mainly were involved in proteolysis (16 genes, 27.78%), protein synthesis (12 genes, 22%), ATP synthesis-coupled proton transport (8 genes, 14.81%), electron transport and ATP biosynthesis. The subcellular distribution pattern of the differentially expressed genes was also analyzed. As shown in Fig. 2b, the ribosomes (10 genes, 23.81%), proton-transporting two-sector ATPase complex (8 genes, 19.5%), intracellular (11 genes, 26.19%) and membrane (7 genes, 16.67%) were the main cellular components involved (Fig. 2b).

**Fig. 3:**
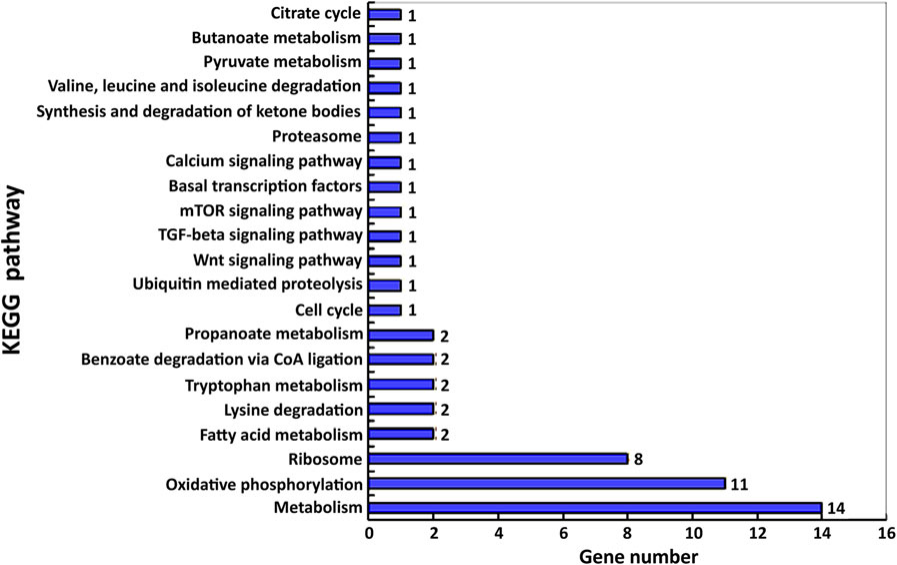
KEGG classification of non-redundant unigenes for *Bombyx mori* Suppression Subtractive Hybridization libraries

KEGG pathway analysis revealed that the differentially expressed genes were involved in fatty acid metabolism, polyketide sugar unit biosynthesis, oxidative phosphorylation, streptomycin biosynthesis, propionate metabolism, β-pyruvate metabolism, and degradation of valine, leucine, isoleucine, and isoleucine (Fig. 3). Relatively more genes were involved in metabolism, oxidative phosphorylation, mitochondria- or ubiquitin-mediated proteolysis and fatty acid metabolism.

### Quantitative real-time PCR

**Fig. 4:**
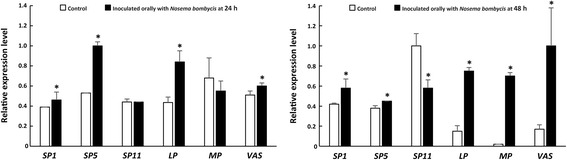
Quantitative real-time PCR analysis for six genes expressed in the midgut of *Bombyx mori*. The expression levels of selected genes were upregulated at the time points of 24 and 48 hours in the midgut of *Bombyx mori* infected by *Nosema bombycis*. *Abbreviations*: *sp*1: serine protease 1; *sp*5: serine protease 5; *sp*11: serine protease 11; *lp*: lipase; *mp*: membrane protein; *vas*: vacuolar ATP synthase subunit G. Data are presented as the mean ± SE of triplicate experiments. Statistically significant differences between the infected groups and control non-infected groups were determined using Student’s t-test. *P-value of ≤ 0.05 was considered as statistically significant

To validate the changes in mRNA abundance detected by SSH and analyze the transcriptional responses of the silkworm to *N. bombycis* infection, qPCR was performed on 6 differentially expressed genes, including serine protease 1 (*sp1*, accession number NM_001043361), serine protease 5 (*sp5*, accession number AAX39408.1), serine protease 11 (*sp11*, accession number JX312360.1), lipase (*lp*, accession number XM_012695610.2), membrane protein (*mp*, accession number XM_021353371.1), and vacuolar ATP synthase subunit G (*vas*, accession number NM_001046822.1). As shown in Fig. 4, these genes were significantly upregulated 48 hours after *N. bombycis* infection except for *sp11* at 24 hours, consistent with the results from the subtracted cDNA libraries-based investigation (Additional file 1: Table S1). The results of qPCR further demonstrated that the subtractive hybridization library was successfully constructed and also suggested that these genes may be involved in the interaction between *N. bombycis* and the host *B. mori*.

### Total protein, fatty acid and ATP contents in midgut tissue

**Fig. 5:**
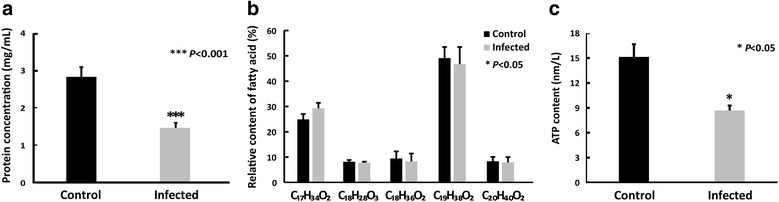
Analysis of the total protein, fatty acid and ATP contents in the midgut of *Bombyx mori*. **a** Total protein concentration of midgut tissues of *B. mori* infected with *N. bombycis* and uninfected. **b** The relative content of fatty acid in infected and uninfected midgut tissue of *B. mori*; C_17_H_34_O_2_: hexadecanoic acid methyl esters; C_18_H_28_O_3_: benzenepropanoic acid, 3,5-bis(1,1-dimethylethyl)-4-hydroxy-, methyl ester); C_18_H_36_O_2_: palmitic acid ethyl ester; C_19_H_38_O_2_: methyl stearate; C_20_H_40_O_2_: octadecanoic acid ethyl ester; **c** The content of ATP in infected and uninfected midgut tissue of *B. mori*. Data are presented as the mean ± SE of triplicate experiments. Statistically significant differences between the infected groups and control non-infected groups were determined using Student’s t-test. *P-value of ≤ 0.05 was considered as statistically significant

To evaluate whether nutrients materials and energy in the midgut of *B. mori* change in response to *N. bombycis* infection, the content of protein, fatty acid and ATP in the midgut tissues was determined. GC-MS analysis results showed that the fatty acids in *B. mori* midgut mainly consisted in C_17_H_34_O_2_ (hexadecanoic acid methyl esters), C_18_H_28_O_3_ (benzenepropanoic acid, 3,5-bis(1,1-dimethylethyl)-4-hydroxy-, methyl ester), C_18_H_36_O_2_ (palmitic acid ethyl ester), C_19_H_38_O_2_ (methyl stearate), and C_20_H_40_O_2_ (octadecanoic acid ethyl ester) (Fig. 5b, Additional file 2: Table S2). No significant changes in fatty acids content after 4 days of *N. bombycis* infection were observed compared with the control non-infected groups (Fig. 5b). However, it is worth noting that the total protein and ATP contents were significantly decreased due to *N. bombycis* infection (Fig. 5a, c).

## Discussion

The complex interaction between pathogens and insects begins in the midgut cells [26, 27]. Many studies have observed that pathogenic microsporidia *N. bombycis*, cytoplasmic polyhedrosis virus (CPV), *Bombyx mori* nucleopolyhedro virus (*BmNPV*), *Escherichia coli*, *Bacillus bombyseptieus*, *Beauveria bassiana* and *Bacillus thuringiensis* infections promote the expression of immune-related genes [11–13]. However, there is little information available of how microsporidia obtain the material and energy from host cells. The microsporidia successfully enter the host midgut cells where the spores initiate the first-round proliferation. In this stage, similar to other intracellular pathogens, the spores need nutrients and energy from host cells to meet for proliferation. However, since microsporidia lack mitochondria, and their tricarboxylic acid (TCA) cycle and oxidative phosphorylation pathways for ATP production have been lost [2, 5, 9, 10, 26–28], the energy generated by the glycolysis pathway is insufficient to facilitate their growth and reproduction. Therefore, additional energy needs to be obtained from the host cells. One study speculated that microsporidia *Encephalitozoon cuniculi* might use NTT proteins to exploit the ATP pool of its eukaryotic host to complete their life-cycle [29].

In this study, we performed SSH and qPCR to investigate gene expression patterns in the midgut of *B. mori* infected with the microsporidian *N. bombysis* and paid particular attention to genes related to energy metabolism, fat and protein degradation. SSH analysis showed that eight genes related to proton-coupled ATP synthesis and 11 genes involved in oxidative phosphorylation of *B. mori* were significantly upregulated after 48 h of *N. bombycis* infection. These results indicate that *N. bombycis* invasion resulted in upregulation of the expression levels of host genes related to ATP synthesis, which might significantly increase ATP production to ensure *N. bombycis* energy requirements. Interestingly, in this study, SSH analysis indicated that the expression level of succinyl coenzyme A synthetase (XM_004928937.3), a key enzyme in the TCA cycle of the host midgut, was significantly increased, suggesting that ATP production in the host was enhanced through the TCA cycle [30, 31]. However, biochemical analysis indicated that the ATP content in the infected midgut tissue did not significantly increase as expected. Instead, the amount of ATP content was significantly decreased compared with the uninfected midgut. Given the expression level of those ATP synthesis related genes in host midgut were significantly upregulated (Fig 2a), this result implied the existence of an additional energy consumer during this procedure. The *N. bombycis* might act this role elevating the total energy expenditure and sharing the increased ATP sythesized by *B. mor*i, which is required by the extra energy demand of its specific parasitic life.

As an obligate intracellular parasite with a highly reduced and compacted genome, the microsporidian *N. bombycis* has lost some genes involved in material metabolism [32, 33]. For example, *N. bombycis* lacks a large number of genes related to the *de novo* synthesis of amino acids [6]. Most intracellular pathogens utilize fatty acids as carbon sources to cope with nutrient deprivation during the infection process, and the microsporidian *N. bombycis* may be no exception. *Nosema bombycis* may need protein and fatty acids from the host to ensure the completion of the life-cycle. Interestingly, in this study, transcriptome data showed that protein degradation related genes in *B. mori* were significantly upregulated following *N. bombycis* invasion. The ubiquitin-proteasome system (UPS) is the major intracellular protein degradation pathway. *Nosema bombycis* infection leads to the elevated expression of protein degradation related enzyme in the host midgut, including the S-phase kinase-associated protein 1 (NP_001040518.1) and the ubiquitin-conjugating enzyme E2G (XP_973689.1) both of which are involved in the ubiquitin-proteasome pathway. In addition, serine proteases are involved in protein degradation. Some serine protease genes were also identified in our subtractive library. Fortunately, the molecular evidence was also eventually supported by the biochemical data that the protein content of infected midgut tissue was significantly decreased compared with the uninfected midgut. In addition, fat degradation related genes in *B. mori* were also significantly upregulated due to *N. bombycis* invasion, including lipase-1 (NP_001036966), fatty acyl-coenzyme A dehydrogenase (NP_001086623.1), and acetyl coenzyme A thiolase (NP_001093296.1). Among these, the expression level of lipase-1 was particularly high (Fig. 4), indicating that *N. bombycis* infection accelerated fat hydrolysis and fatty acid β-oxidation in *B. mori* midgut cells. Moreover, because *N. bombycis* lacks the pathways for *de novo* synthesis of fatty acids but retains a fatty acid transporter gene and long-chain fatty acid CoA ligase gene [6], we speculate that *N. bombycis* could obtain short-chain fatty acids from the host cells to synthesize long-chain fatty acids. This would result in significant changes in fatty acids content in the host midgut. Unfortunately, further GC-MS analysis showed that the fatty acids content of infected midgut did not change significantly compared with the uninfected groups. Based on this result, we speculate that *N. bombycis* prefer obtaining proteins over fatty acids from the host to ensure the material requirements for parasitic life.

Given that *N. bombycis* infection leads to elevated expression of host proteins, fat degradation and ATP synthesis related gene, and a significant decline of protein and ATP content in host midgut, we believe that the amino acids and ATP needed for *N. bombycis* growth and reproduction are subtracted from the host, rather than being synthetized *de novo*. The advantage of subtracting amino acids and ATP from the host is that *N. bombycis* can quickly acquire large amounts of materials and energy required for its life-cycle. However, the disadvantage of this strategy is also apparent: as the extended progression of the infection duration, the catabolism rate of proteins and ATP is about to surpass their synthesis rate, which would result in the damage and eventual death of the host cells. Therefore, keeping an appropriate rate for the utilization of proteins and ATP from the host cells is important for the parasitic life of *N. bombycis.*

## Conclusions

SSH, qPCR, and ATP, protein and fatty acids content analysis suggest that *N. bombycis* invasion can activate host protein degradation and potentially accelerate host ATP production, to ensure enough nutrients and energy for the completion of the parasite life-cycle. This study enriches our understanding of how the microsporidia *N. bombycis* obtain energy and material from host cells, and provides valuable information for future functional investigations of key molecules responsible for energy and material transfer from *B. mori* to *N. bombycis*, to use as a basis for the identification of drug target candidates.

## Additional files

Additional file 1: **Table S1.** The differently expressed genes in the SSH cDNA library. A total of 110 differentially expressed genes in the midgut of *Bombyx mori* during the early stage of *Nosema bombycis* infection were systemically identified by suppression subtractive hybridization (SSH). (DOCX 29 kb)

Additional file 2: **Table S2.** The relative content of fatty acids in control and infection midgut tissue of *Bombyx mori*. Fatty acid quantitative data for midgut tissue of *B. mori*. Results expressed in area % of fatty acid in the sample. (DOCX 71 kb)

## Abbreviations

*BmNPV*: *Bombyx mori* nucleopolyhedro virus
CPV: cytoplasmic polyhedrosis virus
ESTs: expressed sequence tags
GO: Gene Ontology
KEGG: Kyoto Encyclopedia of Genes and Genomes
*lp*: lipase
*mp*: membrane protein
qPCR: quantitative real-time PCR
*sp*1: serine protease 1
*sp*11: serine protease 11
*sp*5: serine protease 5
SSH: suppression subtractive hybridization
TCA: tricarboxylic acid
UPS: ubiquitin-proteasome system
*vas*: vacuolar ATP synthase subunit G
