# Supplementary material for: Molecular and biochemical responses in the midgut of the silkworm, Bombyx mori, infected with Nosema bombycis
Source: Parasit Vectors. 2018 Mar 6;11:147. doi: 10.1186/s13071-018-2755-2 (PMC5840838; doi:10.1186/s13071-018-2755-2)
Supplement: Supplementary file 1 — Table S1. The differently expressed genes in the SSH cDNA library. A total of 110 differentially expressed genes in the midgut of Bombyx mori during the early stage of Nosema bombycis infection were systemically identified by suppression subtractive hybridization (SSH). (DOCX 29 kb) [file 13071_2018_2755_MOESM1_ESM.docx]

**Table S1. Differentially expressed genes from the SSH library**

| **Accession number in genome** | **GeneBank accession number** | **Annotation** | **E values** |
| --- | --- | --- | --- |
| BGIBMGA000231-PA | NP_001040169.1 | Vacuolar atp synthase 21 kda proteolipid subunit | 2.00E-80 |
| BGIBMGA000395-PA | NP_001037247.1 | Ribosomal protein l37 | 8.00E-48 |
| BGIBMGA000867-PA | NP_001037564.1 | Ribosomal protein s2 | 1.00E-109 |
| BGIBMGA000897-PA | XP_002008276.1 | Nadh-ubiquinone oxidoreductase 39 kda subunit | 2.00E-72 |
| BGIBMGA000918-PA | NP_001040518.1 | S-phase kinase-associated protein 1 | 3.00E-64 |
| BGIBMGA001173-PA | NP_001091744.1 | Alkaline nuclease | 1.00E-128 |
| BGIBMGA001302-PA | ADX36413.1 | Novel sequence | 7.00E-09 |
| BGIBMGA001302-PA | NP_001091762.1 | Vacuolar h | 3.00E-60 |
| BGIBMGA001320-PA | ADA83702.1 | Serine protease 31 | 4.00E-17 |
| BGIBMGA001501-PA | NP_001037566.1 | Ribosomal protein s6 | 7.00E-41 |
| BGIBMGA001508-PA | BAD22559.1 | Pancreatic lipase-like protein | 4.00E-69 |
| BGIBMGA001597-PA | XP_001746063.1 | Hypothetical protein | 6.00E-07 |
| BGIBMGA001597-PA |  | Novel sequence | 3.00E-39 |
| BGIBMGA001853-PA | NP_001040233.1 | Mitochondrial h+-transporting atp synthase f1 complex alpha subunit 1 | 1.00E-106 |
| BGIBMGA001916-PA | NP_001040358.1 | Embryonic development factor | 1.00E-109 |
| BGIBMGA002164-PA | NP_001037243.1 | Ribosomal protein l35a | 2.00E-86 |
| BGIBMGA002233-PA |  | Novel sequence | 3.00E-08 |
| BGIBMGA002640-PA | ADQ89804.1 | Ubiquitin carboxyl-terminal hydrolase isozyme l3 | 3.00E-95 |
| BGIBMGA002669-PA | ACB54946.1 | Pancreatic triacylglycerol lipase | 3.00E-78 |
| BGIBMGA002670-PA | NP_001036966.1 | Lipase | 1.00E-139 |
| BGIBMGA002905-PA | NP_001037023.1 | Acyl- -binding protein | 1.00E-42 |
| BGIBMGA002905-PA | NP_001037023.1 | Acyl-CoA binding protein-like | 2.00E-09 |
| BGIBMGA002920-PA | AAF90148.1AF274022_1 | Tetraspanin 29fb | 1.00E-82 |
| BGIBMGA002980-PA | EFA03967.1 | Isoform a | 3.00E-42 |
| BGIBMGA003004-PA | NP_001091757.1 | Small nuclear ribonucleoprotein polypeptide | 6.00E-64 |
| BGIBMGA003166-PA | XP_623868.1 | General transcription factor iif subunit 2-like | 8.00E-37 |
| BGIBMGA003197-PA | NP_001037013.2\| | Ribosomal protein s18 | 8.00E-82 |
| BGIBMGA003322-PA | ABK23569.1 | Membrane protein | 5.00E-28 |
| BGIBMGA003323-PA | ACL99854.1 | Membrane protein | 1.00E-112 |
| BGIBMGA003567-PA | ZP_06799802.1 | Hypothetical protein | 3.00E-12 |
| BGIBMGA003567-PA | AAB26023.1 | Serine protease | 1.00E-104 |
| BGIBMGA003567-PA | ACR15970.1 | Trypsin iib precursor | 2.00E-40 |
| BGIBMGA003567-PA | AAB26023.1 | Trypsin-like serine protease | 4.00E-99 |
| BGIBMGA003567-PA | NP_001040440.1 | Ubiquinone biosynthesis protein coq4 mitochondrial-like | 3.00E-77 |
| BGIBMGA003568-PA | P35045.1TRYA_MANSE | Trypsin-like serine protease | 7.00E-84 |
| BGIBMGA003608-PA | CBI70558.1 | Elongation-factor 1 alpha | 6.00E-18 |
| BGIBMGA003829-PA | NP_001037072.1 | Adp atp translocase | 4.00E-82 |
| BGIBMGA004006-PA | XP_002429263.1 | Cuticle protein cph45 | 2.00E-17 |
| BGIBMGA004006-PA | XP_002429263.1 | Hypothetical protein | 4.00E-17 |
| BGIBMGA004011-PA | ZP_00787405.1 | Cell wall surface anchor family protein | 4.00E-16 |
| BGIBMGA004124-PA | ACY06932.1 | Cuticle protein cph45 | 7.00E-27 |
| BGIBMGA004165-PA | NP_001037593.1 | Translation elongation factor 2 | 5.00E-42 |
| BGIBMGA004288-PA | NP_001136225.1 | Uncharacterized protein loc100216500 precursor | 2.00E-59 |
| BGIBMGA004289-PA | NP_001136225.1 | Uncharacterized protein loc100216500 precursor | 4.00E-26 |
| BGIBMGA004291-PA | NP_001136225.1 | Uncharacterized protein loc100216500 precursor | 3.00E-38 |
| BGIBMGA004292-PA | NP_001136225.1 | Uncharacterized protein loc100216500 precursor | 7.00E-40 |
| BGIBMGA004294-PA | ABH10141.1 | Hmg176 isoform d | 8.00E-08 |
| BGIBMGA004294-PA |  | Novel sequence | 3.00E-60 |
| BGIBMGA004294-PA | NP_001136225.1 | Venom protein r-like protein | 1.00E-10 |
| BGIBMGA004406-PA | ADO33040.1 | Ribosome-associated membrane protein | 3.00E-15 |
| BGIBMGA004488-PA | XP_001660686.1 | Nadh dehydrogenase | 3.00E-39 |
| BGIBMGA005024-PA | ABX39543.1 | Single domain major allergen protein | 3.00E-71 |
| BGIBMGA005302-PA | ACY06938.1 | Cuticular protein hypothetical 43 | 9.00E-33 |
| BGIBMGA005444-PA | NP_001086623.1 | Glutaryl- mitochondrial | 2.00E-54 |
| BGIBMGA005455-PA | NP_001093285.1 | Coiled-coil-helix-coiled-coil-helix domain-containing protein mitochondrial-like | 2.00E-65 |
| BGIBMGA005576-PA | BAJ12014.1 | Actin | 1.00E-112 |
| BGIBMGA005648-PA | CBH09270.1 | Mitochondrial hydrogen-transporting atp synthase coupling factor f | 8.00E-56 |
| BGIBMGA005766-PA | ACY06935.1 | Cuticle protein cph40 | 8.00E-26 |
| BGIBMGA005954-PA | XP_002006742.1 | Cg12926-pa-like protein | 4.00E-28 |
| BGIBMGA005994-PA | NP_001040543.1 | 26s proteasome non-atpase regulatory subunit 7 | 1.00E-86 |
| BGIBMGA006412-PA | Q9U505.1ATP9_MANSE | Atp synthase lipid-binding mitochondrial precursor | 8.00E-47 |
| BGIBMGA006751-PA | ACN67046.1 | Gtp-binding nuclear protein ran | 8.00E-81 |
| BGIBMGA006859-PA | EFR29452.1 | Mitochondrial nadh:ubiquinone oxidoreductase esss | 6.00E-32 |
| BGIBMGA006986-PA | NP_001037225.1 | Ribosomal protein l22 | 9.00E-52 |
| BGIBMGA007710-PA | ACY95347.1 | Ribosomal protein s5 | 2.00E-85 |
| BGIBMGA008050-PA | EFN88033.1 | Myophilin-like isoform 1 | 1.00E-34 |
| BGIBMGA008059-PA | NP_001037013.1 | Aminopeptidase n | 2.00E-96 |
| BGIBMGA008280-PA | CAL92020.1 | Chymotrypsin 1 | 1.00E-86 |
| BGIBMGA008670-PA | NP_001040232.1 | Atp synthase | 1.00E-114 |
| BGIBMGA008780-PA | NP_001037584.1 | Iron storage protein | 1.00E-105 |
| BGIBMGA008861-PA | NP_001040443.1 | Troponin c 25d | 7.00E-80 |
| BGIBMGA008881-PA | NP_001037215.1 | 60s ribosomal protein l11 | 1.00E-105 |
| BGIBMGA009319-PA | ADT80643.1 | Ribosomal protein s3 | 1.00E-54 |
| BGIBMGA009477-PA | CAA06419.1 | Carboxypeptidase a | 2.00E-57 |
| BGIBMGA009671-PA | NP_001040541.1 | Isochorismatase domain containing 1 | 2.00E-33 |
| BGIBMGA009698-PA | NP_001040365.1 | Nascent polypeptide associated complex protein alpha subunit | 8.00E-92 |
| BGIBMGA010024-PA | NP_001093273.1 | Trypsin-like serine protease | 1.00E-128 |
| BGIBMGA010032-PA | XP_001814337.1 | Protein jagunal | 1.00E-13 |
| BGIBMGA010409-PA | XP_001659123.1 | Calponin transgelin | 3.00E-31 |
| BGIBMGA010620-PA | NP_001040390.1 | Syntaxin 5a | 9.00E-62 |
| BGIBMGA010668-PA | NP_001139709.1 | Barrier-to-autointegration factor b | 2.00E-20 |
| BGIBMGA010751-PA |  | Novel sequence | 4.00E-16 |
| BGIBMGA010979-PA | ACL99854.1 | Membrane protein | 3.00E-39 |
| BGIBMGA011029-PA | NP_001093296.1 | Acetoacetyl- thiolase | 2.00E-84 |
| BGIBMGA011504-PA | NP_001040526.1 | h+ transporting atp synthase o subunit isoform 1 | 8.00E-88 |
| BGIBMGA012153-PA | NP_001091812.1 | h+ transporting atp synthase subunit e | 9.00E-38 |
| BGIBMGA012320-PA | XP_973689.1 | Ubiquitin-conjugating enzyme e2 g2 | 3.00E-82 |
| BGIBMGA012437-PA | NP_001166195.1 | Coatomer subunit epsilon | 6.00E-51 |
| BGIBMGA012546-PA | NP_001037231.1 | Ribosomal protein L24 | 1.00E-22 |
| BGIBMGA012777-PA | ACR15967.2\| | Trypsin-like serine proteinase t26 | 2.00E-68 |
| BGIBMGA012780-PA | ADO33055.1 | Succinyl- synthetase small | 1.00E-102 |
| BGIBMGA012788-PA | BAA77401.1 | Trypsin-like serine proteinase t26 | 5.00E-23 |
| BGIBMGA012792-PA | NP_001037037.1 | 35kda protease precursor | 1.00E-18 |
| BGIBMGA012864-PA | BAF03520.1 | Peptidoglycan recognition protein | 1.00E-63 |
| BGIBMGA012890-PA | NP_001037574.1 | Cellular retinoic acid binding protein | 2.00E-52 |
| BGIBMGA012981-PA | XP_971914.1 | Transmembrane emp24 domain-containing protein 2 | 2.00E-59 |
| BGIBMGA013566-PA | NP_001037094.1 | Isoform b | 1.00E-82 |
| BGIBMGA013678-PA | NP_001040287.1 | Vacuolar atp synthase subunit g | 3.00E-22 |
| BGIBMGA013680-PA | NP_001073120.1 | Cytochrome c oxidase polypeptide iv | 4.00E-45 |
| BGIBMGA013930-PA | BAF81491.1 | Juvenile hormone epoxide hydrolase | 9.00E-73 |
| BGIBMGA013964-PA | NP_001040138.1 | Vacuolar h | 2.00E-94 |
| BGIBMGA014094-PA | NP_001037159.1 | Ribosomal protein l14 | 7.00E-19 |
| BGIBMGA014175-PA | ABR27900.1 | Mitochondrial cytochrome c oxidase subunit 6c | 8.00E-18 |
| BGIBMGA014427-PA | NP_001036826.1 | Serine protease | 7.00E-67 |
| BGIBMGA014428-PA | NP_001036826.1 | Serine protease | 1.00E-132 |
| BGIBMGA014429-PA | AAX39408.1 | Serine protease | 1.00E-126 |
| BGIBMGA014429-PA | AAX39408.1 | Serine protease | 6.00E-94 |
| BGIBMGA014431-PA | NP_001036903.1 | Serine protease | 1.00E-132 |
| BGIBMGA014483-PA | XP_966588.1 | Nadh dehydrogenase iron-sulfur protein mitochondrial | 1.00E-100 |
| BGIBMGA014524-PA | NP_001036826.1 | Chymotrypsin-like proteinase | 6.00E-61 |
