# Supplementary material for: Molecular and biochemical responses in the midgut of the silkworm, Bombyx mori, infected with Nosema bombycis
Source: Parasit Vectors. 2018 Mar 6;11:147. doi: 10.1186/s13071-018-2755-2 (PMC5840838; doi:10.1186/s13071-018-2755-2)
Supplement: Supplementary file 2 — Table S2. The relative content of fatty acids in control and infection midgut tissue of Bombyx mori. Fatty acid quantitative data for midgut tissue of B. mori. Results expressed in area % of fatty acid in the sample. (DOCX 71 kb) [file 13071_2018_2755_MOESM2_ESM.docx]

**Table S2. Relative content of fatty acids in control and infection midgut tissue of *Bombyx mori*.**

| **Control-1 midgut tissue** | |  | |  | |  | |  | |  | |  | |  |  |  |  |  |  |
| --- | --- | --- | --- | --- | --- | --- | --- | --- | --- | --- | --- | --- | --- | --- | --- | --- | --- | --- | --- |
| PEAK LIST |  |  | |  | |  | |  | |  | |  | |  |  |  |  |  |  |
| **CAS#** | **Name** | **Formula** | | **Exact Mass** | | **Match** | | **Rmatch** | | **Prob%** | | **Apex RT** | | **Start RT** | **End RT** | **Area** | **%Area** | **Height** | **%Height** |
| 112-39-0 | Hexadecanoic acid, methyl ester | C17H34O2 | | 270.25588 | | 869 | | 898 | | 61 | | 28.11 | | 28.06 | 28.2 | 46708628.33 | 21.99689808 | 13721943.71 | 1.46 |
| 6386-38-5 | Benzenepropanoic acid, 3,5-bis(1,1-dimethylethyl)-4-hydroxy-, methyl ester | C18H28O3 | | 292.203844 | | 808 | | 824 | | 93.6 | | 28.4 | | 28.33 | 28.5 | 19120565.1 | 9.004612993 | 5703639.296 | 0.61 |
| 628-97-7 | Palmitic acid ethyl ester | C18H36O2 | | 284.27153 | | 710 | | 769 | | 50 | | 29.4 | | 29.36 | 29.44 | 2640524.177 | 1.243524875 | 836498.932 | 0.09 |
| 112-61-8 | Methyl stearate | C19H38O2 | | 298.28718 | | 922 | | 926 | | 72.9 | | 31.81 | | 31.76 | 31.85 | 133231660.8 | 62.74393766 | 45316426.09 | 4.82 |
| 111-61-5 | Octadecanoic acid, ethyl ester | C20H40O2 | | 312.30283 | | 655 | | 842 | | 32.8 | | 33.03 | | 32.95 | 33.21 | 10640507.98 | 5.011026397 | 1430321.143 | 0.15 |
|  |  |  | |  | |  | |  | |  | |  | |  |  |  |  |  |  |
|  |  |  | |  | |  | |  | |  | |  | |  |  |  |  |  |  |
| **Control-2 midgut tissue** | |  | |  | |  | |  | |  | |  | |  |  |  |  |  |  |
| PEAK LIST |  |  | |  | |  | |  | |  | |  | |  |  |  |  |  |  |
| **CAS#** | **Name** | **Formula** | | **Exact Mass** | | **Match** | | **Rmatch** | | **Prob%** | | **Apex RT** | | **Start RT** | **End RT** | **Area** | **%Area** | **Height** | **%Height** |
| 112-39-0 | Hexadecanoic acid, methyl ester | C17H34O2 | | 270.25588 | | 887 | | 894 | | 64.3 | | 28.11 | | 28.05 | 28.2 | 125040286.5 | 28.4511315 | 38889300.85 | 0.72 |
| 6386-38-5 | Benzenepropanoic acid, 3,5-bis(1,1-dimethylethyl)-4-hydroxy-, methyl ester | C18H28O3 | | 292.203844 | | 771 | | 786 | | 95.2 | | 28.4 | | 28.33 | 28.5 | 32097179.78 | 7.303254877 | 9128107.558 | 0.17 |
| 628-97-7 | Palmitic acid ethyl ester | C18H36O2 | | 284.27153 | | 718 | | 778 | | 47.1 | | 29.4 | | 29.33 | 29.44 | 9138840.215 | 2.079412579 | 2573472.418 | 0.05 |
| 112-61-8 | Methyl stearate | C19H38O2 | | 298.28718 | | 892 | | 896 | | 74.6 | | 31.82 | | 31.77 | 31.86 | 265042377.8 | 60.306608 | 93007862.97 | 1.72 |
| 111-61-5 | Octadecanoic acid, ethyl ester | C20H40O2 | | 312.30283 | | 616 | | 653 | | 67.7 | | 33.03 | | 32.96 | 33.08 | 8172752.223 | 1.859593053 | 2164170.961 | 0.04 |
|  |  |  | |  | |  | |  | |  | |  | |  |  |  |  |  |  |
|  |  |  | |  | |  | |  | |  | |  | |  |  |  |  |  |  |
| **Control-3 midgut tissue** | |  | |  | |  | |  | |  | |  | |  |  |  |  |  |  |
| PEAK LIST |  |  | |  | |  | |  | |  | |  | |  |  |  |  |  |  |
| **CAS#** | **Name** | **Formula** | | **Exact Mass** | | **Match** | | **Rmatch** | | **Prob%** | | **Apex RT** | | **Start RT** | **End RT** | **Area** | **%Area** | **Height** | **%Height** |
| 112-39-0 | Hexadecanoic acid, methyl ester | C17H34O2 | | 270.25588 | | 900 | | 904 | | 63.9 | | 28.1 | | 28.04 | 28.2 | 49292553.13 | 30.44656449 | 17542853.73 | 3.43 |
| 6386-38-5 | Benzenepropanoic acid, 3,5-bis(1,1-dimethylethyl)-4-hydroxy-, methyl ester | C18H28O3 | | 292.203844 | | 788 | | 802 | | 95.7 | | 28.39 | | 28.33 | 28.49 | 12276604.31 | 7.582898451 | 3929830.761 | 0.77 |
| 628-97-7 | Palmitic acid ethyl ester | C18H36O2 | | 284.27153 | | 871 | | 876 | | 57 | | 29.4 | | 29.34 | 29.5 | 22040436.62 | 13.61373133 | 7068247.163 | 1.38 |
| 112-61-8 | Methyl stearate | C19H38O2 | | 298.28718 | | 892 | | 896 | | 78.1 | | 31.8 | | 31.75 | 31.85 | 58631506.51 | 36.21496212 | 20347809.26 | 3.97 |
| 111-61-5 | Octadecanoic acid, ethyl ester | C20H40O2 | | 312.30283 | | 646 | | 652 | | 62.1 | | 32.97 | | 32.9 | 33.09 | 19657471.41 | 12.14184361 | 5991394.095 | 1.17 |
|  |  |  | |  | |  | |  | |  | |  | |  |  |  |  |  |  |
|  |  |  | |  | |  | |  | |  | |  | |  |  |  |  |  |  |
| **Control-4 midgut tissue** | |  | |  | |  | |  | |  | |  | |  |  |  |  |  |  |
| PEAK LIST |  |  | |  | |  | |  | |  | |  | |  |  |  |  |  |  |
| **CAS#** | **Name** | **Formula** | | **Exact Mass** | | **Match** | | **Rmatch** | | **Prob%** | | **Apex RT** | | **Start RT** | **End RT** | **Area** | **%Area** | **Height** | **%Height** |
| 112-39-0 | Hexadecanoic acid, methyl ester | C17H34O2 | | 270.25588 | | 902 | | 905 | | 65.5 | | 28.1 | | 28.04 | 28.3 | 54369174.85 | 35.51242222 | 16799425.74 | 3.31 |
| 6386-38-5 | Benzenepropanoic acid, 3,5-bis(1,1-dimethylethyl)-4-hydroxy-, methyl ester | C18H28O3 | | 292.203844 | | 763 | | 773 | | 93.5 | | 28.39 | | 28.33 | 28.48 | 10241764.48 | 6.689633704 | 3360849.4 | 0.66 |
| 628-97-7 | Palmitic acid ethyl ester | C18H36O2 | | 284.27153 | | 874 | | 877 | | 60 | | 29.41 | | 29.34 | 29.51 | 26296215.34 | 17.17595135 | 8112919.829 | 1.6 |
| 112-61-8 | Methyl stearate | C19H38O2 | | 298.28718 | | 879 | | 897 | | 74.8 | | 31.8 | | 31.75 | 31.84 | 42279807.36 | 27.61598598 | 14788459.58 | 2.92 |
| 111-61-5 | Octadecanoic acid, ethyl ester | C20H40O2 | | 312.30283 | | 842 | | 860 | | 52.1 | | 32.98 | | 32.91 | 33.08 | 19912070.49 | 13.00600674 | 6023158.803 | 1.19 |
|  |  |  | |  | |  | |  | |  | |  | |  |  |  |  |  |  |
|  |  |  | |  | |  | |  | |  | |  | |  |  |  |  |  |  |
| **Control-5 midgut tissue** | |  | |  | |  | |  | |  | |  | |  |  |  |  |  |  |
| PEAK LIST |  |  | |  | |  | |  | |  | |  | |  |  |  |  |  |  |
| **CAS#** | **Name** | **Formula** | | **Exact Mass** | | **Match** | | **Rmatch** | | **Prob%** | | **Apex RT** | | **Start RT** | **End RT** | **Area** | **%Area** | **Height** | **%Height** |
| 112-39-0 | Hexadecanoic acid, methyl ester | C17H34O2 | | 270.25588 | | 881 | | 908 | | 61.4 | | 28.11 | | 28.05 | 28.2 | 44534408.76 | 29.9071689 | 14325926.22 | 2.5 |
| 6386-38-5 | Benzenepropanoic acid, 3,5-bis(1,1-dimethylethyl)-4-hydroxy-, methyl ester | C18H28O3 | | 292.203844 | | 785 | | 862 | | 94.1 | | 28.39 | | 28.32 | 28.49 | 12583584.29 | 8.450530527 | 3948329.113 | 0.69 |
| 628-97-7 | Palmitic acid ethyl ester | C18H36O2 | | 284.27153 | | 806 | | 817 | | 68.6 | | 29.4 | | 29.33 | 29.45 | 11012273.9 | 7.395313974 | 3705347.974 | 0.65 |
| 112-61-8 | Methyl stearate | C19H38O2 | | 298.28718 | | 876 | | 880 | | 76.2 | | 31.81 | | 31.76 | 31.85 | 69449097.91 | 46.63867691 | 22919929.11 | 4 |
| 111-61-5 | Octadecanoic acid, ethyl ester | C20H40O2 | | 312.30283 | | 567 | | 594 | | 51.4 | | 32.99 | | 32.91 | 33.19 | 11329443.27 | 7.608309687 | 2021208.666 | 0.35 |
|  |  |  | |  | |  | |  | |  | |  | |  |  |  |  |  |  |
|  |  |  | |  | |  | |  | |  | |  | |  |  |  |  |  |  |
|  |  |  | |  | |  | |  | |  | |  | |  |  |  |  |  |  |
|  |  |  | |  | |  | |  | |  | |  | |  |  |  |  |  |  |
| **Infection-1 midgut tissue** | | |  | |  | |  | |  | |  | |  |  |  |  |  |  |  |
| PEAK LIST |  | |  | |  | |  | |  | |  | |  |  |  |  |  |  |  |
| **CAS#** | **Name** | | **Formula** | | **Exact Mass** | | **Match** | | **Rmatch** | | **Prob%** | | **Apex RT** | **Start RT** | **End RT** | **Area** | **%Area** | **Height** | **%Height** |
| 112-39-0 | Hexadecanoic acid, methyl ester | | C17H34O2 | | 270.25588 | | 915 | | 916 | | 67.7 | | 28.12 | 28.05 | 28.2 | 433077634.3 | 33.34352786 | 138060605.6 | 3.42 |
| 6386-38-5 | Benzenepropanoic acid, 3,5-bis(1,1-dimethylethyl)-4-hydroxy-, methyl ester | | C18H28O3 | | 292.203844 | | 795 | | 803 | | 94.5 | | 28.39 | 28.32 | 28.49 | 81887330.06 | 6.304672085 | 24023280.78 | 0.6 |
| 628-97-7 | Palmitic acid ethyl ester | | C18H36O2 | | 284.27153 | | 894 | | 895 | | 76.9 | | 29.41 | 29.34 | 29.46 | 113430942.1 | 8.733278928 | 35522550.43 | 0.88 |
| 112-61-8 | Methyl stearate | | C19H38O2 | | 298.28718 | | 903 | | 917 | | 72 | | 31.82 | 31.77 | 31.86 | 586174779.5 | 45.13078844 | 189420282.4 | 4.7 |
| 111-61-5 | Octadecanoic acid, ethyl ester | | C20H40O2 | | 312.30283 | | 867 | | 876 | | 68.6 | | 32.98 | 32.93 | 33.11 | 84264986.36 | 6.487732679 | 23209058.86 | 0.58 |
|  |  | |  | |  | |  | |  | |  | |  |  |  |  |  |  |  |
|  |  | |  | |  | |  | |  | |  | |  |  |  |  |  |  |  |
| **Infection-2 midgut tissue** | | |  | |  | |  | |  | |  | |  |  |  |  |  |  |  |
| PEAK LIST |  | |  | |  | |  | |  | |  | |  |  |  |  |  |  |  |
| **CAS#** | **Name** | | **Formula** | | **Exact Mass** | | **Match** | | **Rmatch** | | **Prob%** | | **Apex RT** | **Start RT** | **End RT** | **Area** | **%Area** | **Height** | **%Height** |
| 112-39-0 | Hexadecanoic acid, methyl ester | | C17H34O2 | | 270.25588 | | 828 | | 874 | | 42.2 | | 28.12 | 28.06 | 28.21 | 20840585.4 | 22.9053406 | 5943781.091 | 1.35 |
| 6386-38-5 | Benzenepropanoic acid, 3,5-bis(1,1-dimethylethyl)-4-hydroxy-, methyl ester | | C18H28O3 | | 292.203844 | | 748 | | 833 | | 91 | | 28.4 | 28.34 | 28.49 | 6826539.575 | 7.502870531 | 1945980.131 | 0.44 |
| 628-97-7 | Palmitic acid ethyl ester | | C18H36O2 | | 284.27153 | | 869 | | 874 | | 54.7 | | 29.41 | 29.33 | 29.5 | 18375173.8 | 20.19567139 | 5515587.856 | 1.25 |
| 112-61-8 | Methyl stearate | | C19H38O2 | | 298.28718 | | 903 | | 909 | | 70.6 | | 31.82 | 31.77 | 31.85 | 31194116.76 | 34.28463526 | 10917465.09 | 2.47 |
| 111-61-5 | Octadecanoic acid, ethyl ester | | C20H40O2 | | 312.30283 | | 692 | | 732 | | 71.5 | | 32.99 | 32.91 | 33.1 | 13749288.5 | 15.11148223 | 3742120.113 | 0.85 |
|  |  | |  | |  | |  | |  | |  | |  |  |  |  |  |  |  |
|  |  | |  | |  | |  | |  | |  | |  |  |  |  |  |  |  |
| **Infection-3 midgut tissue** | | |  | |  | |  | |  | |  | |  |  |  |  |  |  |  |
| PEAK LIST |  | |  | |  | |  | |  | |  | |  |  |  |  |  |  |  |
| **CAS#** | **Name** | | **Formula** | | **Exact Mass** | | **Match** | | **Rmatch** | | **Prob%** | | **Apex RT** | **Start RT** | **End RT** | **Area** | **%Area** | **Height** | **%Height** |
| 112-39-0 | Hexadecanoic acid, methyl ester | | C17H34O2 | | 270.25588 | | 842 | | 884 | | 55.3 | | 28.12 | 28.06 | 28.32 | 54011800.35 | 21.44376704 | 13243924 | 1.3 |
| 6386-38-5 | Benzenepropanoic acid, 3,5-bis(1,1-dimethylethyl)-4-hydroxy-, methyl ester | | C18H28O3 | | 292.203844 | | 782 | | 792 | | 92.8 | | 28.39 | 28.33 | 28.5 | 20118440.11 | 7.98742386 | 5821611.142 | 0.57 |
| 628-97-7 | Palmitic acid ethyl ester | | C18H36O2 | | 284.27153 | | 837 | | 848 | | 56.8 | | 29.41 | 29.34 | 29.44 | 8976203.663 | 3.563732721 | 2963378.233 | 0.29 |
| 112-61-8 | Methyl stearate | | C19H38O2 | | 298.28718 | | 923 | | 924 | | 76.2 | | 31.81 | 31.76 | 31.85 | 151789064 | 60.26329997 | 51229211.74 | 5.02 |
| 111-61-5 | Octadecanoic acid, ethyl ester | | C20H40O2 | | 312.30283 | | 495 | | 815 | | 39.6 | | 32.99 | 32.94 | 33.21 | 16980947.46 | 6.741776409 | 2173338.233 | 0.21 |
|  |  | |  | |  | |  | |  | |  | |  |  |  |  |  |  |  |
|  |  | |  | |  | |  | |  | |  | |  |  |  |  |  |  |  |
| **Infection-4 midgut tissue** | | |  | |  | |  | |  | |  | |  |  |  |  |  |  |  |
| PEAK LIST |  | |  | |  | |  | |  | |  | |  |  |  |  |  |  |  |
| **CAS#** | **Name** | | **Formula** | | **Exact Mass** | | **Match** | | **Rmatch** | | **Prob%** | | **Apex RT** | **Start RT** | **End RT** | **Area** | **%Area** | **Height** | **%Height** |
| 112-39-0 | Hexadecanoic acid, methyl ester | | C17H34O2 | | 270.25588 | | 883 | | 893 | | 62.7 | | 28.11 | 28.06 | 28.21 | 66954942.97 | 22.77819999 | 20036776.27 | 2.2 |
| 6386-38-5 | Benzenepropanoic acid, 3,5-bis(1,1-dimethylethyl)-4-hydroxy-, methyl ester | | C18H28O3 | | 292.203844 | | 789 | | 804 | | 90.6 | | 28.39 | 28.32 | 28.5 | 25457805.08 | 8.660794104 | 8057318.45 | 0.88 |
| 628-97-7 | Palmitic acid ethyl ester | | C18H36O2 | | 284.27153 | | 848 | | 851 | | 56.6 | | 29.41 | 29.34 | 29.46 | 24083252.14 | 8.193168559 | 7191728.079 | 0.79 |
| 112-61-8 | Methyl stearate | | C19H38O2 | | 298.28718 | | 927 | | 931 | | 73.8 | | 31.81 | 31.76 | 31.85 | 154025987.6 | 52.39993633 | 54178547.24 | 5.94 |
| 111-61-5 | Octadecanoic acid, ethyl ester | | C20H40O2 | | 312.30283 | | 645 | | 649 | | 39.5 | | 32.98 | 32.92 | 33.11 | 23421093.8 | 7.96790102 | 5200870.651 | 0.57 |
|  |  | |  | |  | |  | |  | |  | |  |  |  |  |  |  |  |
|  |  | |  | |  | |  | |  | |  | |  |  |  |  |  |  |  |
| **Infection-5 midgut tissue** | | |  | |  | |  | |  | |  | |  |  |  |  |  |  |  |
| PEAK LIST |  | |  | |  | |  | |  | |  | |  |  |  |  |  |  |  |
| **CAS#** | **Name** | | **Formula** | | **Exact Mass** | | **Match** | | **Rmatch** | | **Prob%** | | **Apex RT** | **Start RT** | **End RT** | **Area** | **%Area** | **Height** | **%Height** |
| 112-39-0 | Hexadecanoic acid, methyl ester | | C17H34O2 | | 270.25588 | | 865 | | 942 | | 57.7 | | 28.1 | 28.04 | 28.19 | 170088864.8 | 24.04247113 | 53957660.37 | 2.59 |
| 6386-38-5 | Benzenepropanoic acid, 3,5-bis(1,1-dimethylethyl)-4-hydroxy-, methyl ester | | C18H28O3 | | 292.203844 | | 817 | | 830 | | 95.5 | | 28.39 | 28.32 | 28.48 | 73793536.04 | 10.43089424 | 24461591.9 | 1.17 |
| 628-97-7 | Palmitic acid ethyl ester | | C18H36O2 | | 284.27153 | | 867 | | 874 | | 71.8 | | 29.4 | 29.32 | 29.45 | 46084082.37 | 6.514096152 | 14101534.63 | 0.68 |
| 112-61-8 | Methyl stearate | | C19H38O2 | | 298.28718 | | 912 | | 913 | | 74.9 | | 31.81 | 31.75 | 31.84 | 377874744.4 | 53.41350618 | 124472892.8 | 5.97 |
| 111-61-5 | Octadecanoic acid, ethyl ester | | C20H40O2 | | 312.30283 | | 627 | | 641 | | 66.5 | | 32.98 | 32.92 | 33.11 | 39610447.85 | 5.599032305 | 9452959.451 | 0.45 |
|  |  | |  | |  | |  | |  | |  | |  |  |  |  |  |  |  |
| **Note** |  | |  | |  | |  | |  | |  | |  |  |  |  |  |  |  |
| **CAS#:** | **CAS Registry Number** | |  | |  | |  | |  | |  | |  |  |  |  |  |  |  |
| **Apex RT:** | **Apex retention time** | |  | |  | |  | |  | |  | |  |  |  |  |  |  |  |
